# 1411. Operating room air may harbor pathogens: the role of an ultraviolet air filtration unit

**DOI:** 10.1093/ofid/ofad500.1248

**Published:** 2023-11-27

**Authors:** Diana Fernández-Rodríguez, Saad Tarabichi, Krystal Golankiewicz, Nicolina Zappley, Javad Parvizi

**Affiliations:** MD/PhD Plan de Estudios Combinados en Medicina (PECEM), Mexico City, Distrito Federal, Mexico; Rothman Orthopaedic Institute, Philadelphia, Pennsylvania; Rothman Orthopaedic Institute, Philadelphia, Pennsylvania; Rothman Orthopaedic Institute, Philadelphia, Pennsylvania; Rothman Orthopaedic Institute, Philadelphia, Pennsylvania

## Abstract

**Background:**

Prevention of surgical site infections (SSIs) involves implementation of numerous steps including ultraclean air in the operating room (OR). Despite all efforts, particles in the room air may exist and some of these particles may be live pathogens that can potentially cause subsequent SSI. This prospective study aimed to determine and compare the nature and quantity of microbes in the operating room, as detected from the inlet flow of an ultraviolet filtration unit, and the efficacy of the unit to remove particles and creating clean room air (the outlet flow).

**Methods:**

This prospective study was conducted at a single institution, where primary total joint arthroplasty and spine surgeries were performed. The OR was fitted with a positive ventilation system. In addition, a filtration unit with a crystalline ultraviolet unit (C-UVC) was placed in the OR. The inflow and outflow air from the unit was sampled using specialized swabs at the beginning and at conclusion of each procedure. Additional surgical-related variables were also recorded at each time of sampling. Swabs were processed for culture and Next-Generation Sequencing.

Operating room floor plan

**Figure:**
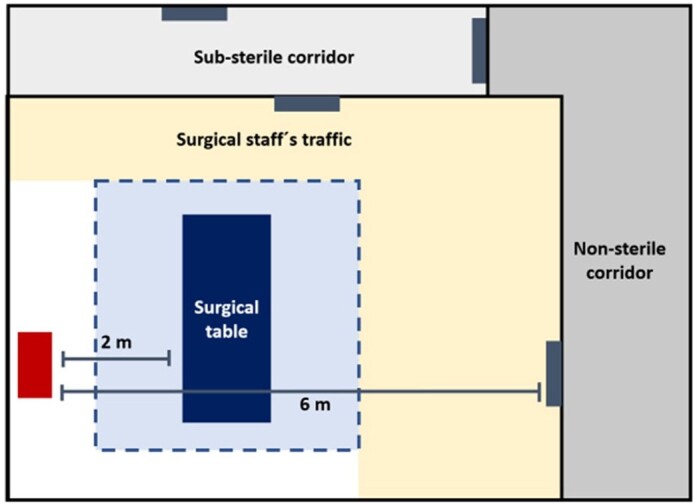

The filtration unit with ultraviolet-C light is represented with a red rectangle and doors (source of contamination) are represented by dark gray rectangles.

Air flow and air swabs collection.

**Figure:**
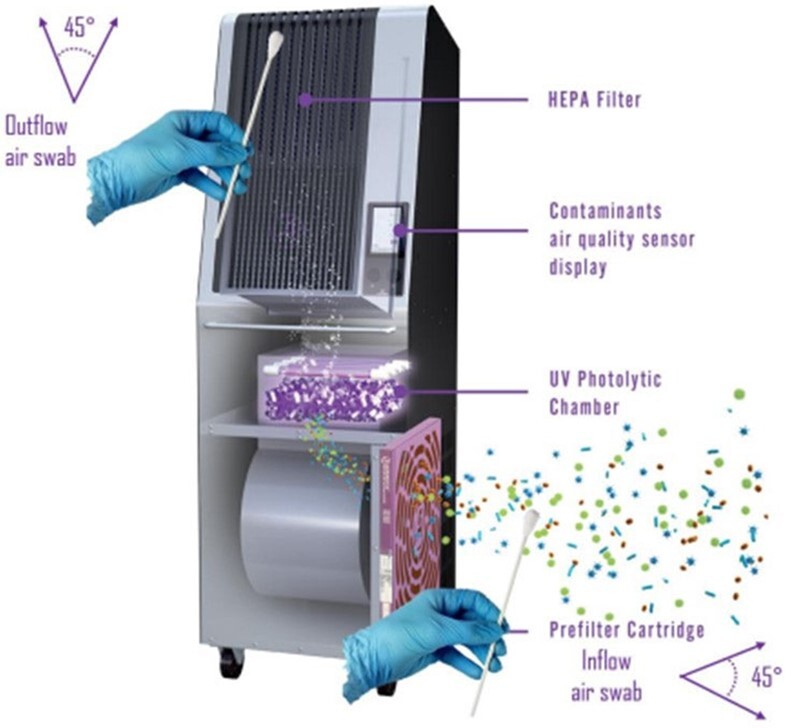

**Results:**

The mean length of the surgical procedures sampled was 68 ± 13 minutes. Overall, 19 out of 200 (9.5%) swabs isolated microorganisms. Inflow swabs were positive at a higher rate (16% vs. 3%; p< 0.01), compared to the outcoming air swabs. A wide variety of Gram-positive, Gram-negative, anaerobic bacteria, and fungi (only in the inflow swabs) were isolated. The detection of microorganisms was also higher in surgical procedures with a higher number of door openings (32.5 ± 7.1 vs. 27.9 ± 5.6; p< 0.01).

Surgical-related variables collected at air swab sampling.

**Figure:**
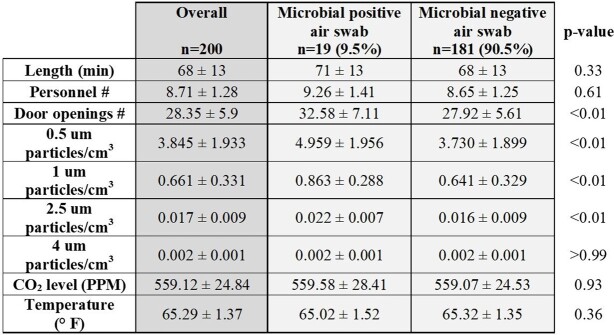

**Conclusion:**

Microorganisms are present in the operating room air. A specialized filtration unit with a C-UVC light was effective in filtering these microorganisms in the majority of cases.

**Disclosures:**

**Javad Parvizi, MD, FRCS**, 3M: Grant/Research Support|Acumed, LLC: Stocks/Bonds|Aesculap: Grant/Research Support|Alphaeon: Stocks/Bonds|AO Spine: Stocks/Bonds|Becton Dickenson: Advisor/Consultant|Biomet: Grant/Research Support|Cardinal Health: Advisor/Consultant|Cempra: Grant/Research Support|CeramTec: Grant/Research Support|Ceribell: Stocks/Bonds|Coracoid: Stocks/Bonds|Corentec: Advisor/Consultant|Datatrace: Grant/Research Support|DePuy: Grant/Research Support|Elsevier: Grant/Research Support|Elute: Stocks/Bonds|Ethicon: Advisor/Consultant|Hip Innovation Technology: Stocks/Bonds|Illuminus: Stocks/Bonds|Integra: Grant/Research Support|Intellijoint: Stocks/Bonds|Jaypee Publishers: Grant/Research Support|KCI / 3M (Acelity): Advisor/Consultant|Lima: Grant/Research Support|MicroGenDx: Advisor/Consultant|Molecular Surface Technologies: Stocks/Bonds|Myoscience: Grant/Research Support|Nanooxygenic: Stocks/Bonds|National Institutes of Health (NIAMS & NICHD): Grant/Research Support|NDRI: Grant/Research Support|Novartis: Grant/Research Support|OREF: Grant/Research Support|Orthospace: Grant/Research Support|Osteal: Stocks/Bonds|Parvizi Surgical Innovations and Subsidiaries: Stocks/Bonds|Peptilogic: Stocks/Bonds|Peptilogics: Advisor/Consultant|Pfizer: Grant/Research Support|PRN-Veterinary: Grant/Research Support|Rotation Medical: Grant/Research Support|Simplify Medical: Grant/Research Support|SLACK Incorporated: Grant/Research Support|Smith & Nephew: Grant/Research Support|Sonata: Stocks/Bonds|Stelkast: Grant/Research Support|Stryker: Grant/Research Support|Synthes: Grant/Research Support|Tenor: Advisor/Consultant|TissueGene: Grant/Research Support|Tornier: Grant/Research Support|Wolters Kluwer Health - Lippincott Williams & Wilkins: Grant/Research Support|Zimmer Biomet: Advisor/Consultant|Zimmer Biomet: Grant/Research Support

